# Characterization of a novel aspartyl protease inhibitor from *Haemonchus contortus*

**DOI:** 10.1186/s13071-017-2137-1

**Published:** 2017-04-19

**Authors:** Baojie Li, Javaid Ali Gadahi, Wenxiang Gao, Zhenchao Zhang, Muhammad Ehsan, Lixin Xu, Xiaokai Song, Xiangrui Li, Ruofeng Yan

**Affiliations:** 1 College of Veterinary Medicine, Nanjing Agricultural University, Nanjing, 210095 People’s Republic of China; 2 Department of Veterinary Parasitology, Sindh Agriculture University, Tandojam, Pakistan

**Keywords:** *Haemonchus contortus*, Aspartyl protease inhibitor, Localization, Differential expression, Inhibitory activity, Induction of cytokines

## Abstract

**Background:**

Aspartyl protease inhibitor (API) was thought to protect intestinal parasitic nematodes from their hostile proteolytic environment. Studies on *Ostertagia ostertagi*, *Ascaris suum* and *Brugia malayi* indicated that aspins might play roles in nematode infection. In a recent study, proteins differentially expressed between free-living third-stage larvae (L3) and activated L3 (xL3) of *Haemonchus contortus* were identified by 2D-DIGE. API was found downregulated in xL3 when compared with L3. However, there was no report about the functions of *H. contortus* API in the parasite-host interaction. In this study, the gene encoding API from *H. contortus* was cloned, expressed, and part of its biological characteristics were studied.

**Results:**

A DNA fragment of 681 bp was amplified by RT-PCR. Ninety one percent of the amino acid sequence was similar with that for aspin from *O. ostertagi*. The recombinant API protein was fusion-expressed with a molecular weight of 48 × 10^3^. Results of Western blot showed that the recombinant API could be recognized by serum from goat infected with *H. contortus*. It was found that API was localized exclusively in the subcutaneous tissue and epithelial cells of the gastrointestinal tract in adult *H. contortus*. qRT-PCR suggested that the API gene was differentially transcribed in different life-cycle stages, with the lowest level in female adults and the highest in free-living L3 larvae. Enzyme inhibition assay indicated that the recombinant API can inhibit the activity of pepsin significantly, and the optimal reaction pH and temperature were 4.0 and 37–50 °C respectively. In vitro study showed that the recombinant API could induce goat PBMCs to express IFN-γ, IL-4 and IL-10.

**Conclusions:**

A new aspartyl protease inhibitor was cloned from *H. contortus* and its characteristics were studied for the first time. The results indicate that API may regulate the immune response of the host and play roles in the infection.

**Electronic supplementary material:**

The online version of this article (doi:10.1186/s13071-017-2137-1) contains supplementary material, which is available to authorized users.

## Background

*Haemonchus contortus* is one of the most important gastrointestinal parasitic nematodes infecting the abomasum of ruminants worldwide, especially sheep, goats and cattle [1]. This nematode feeds on blood and often causes local damage, anemia, and significant production losses, even death in young and weakened hosts, which results in huge economic losses to the livestock industry [2, 3]. This parasite has a life-cycle consisting of free-living stages on pasture (from eggs to L3 larvae) and after ingestion, development through L4 larva to adult in the host gastrointestinal tract [4, 5]. Controlling of *H. contortus* is almost depending on the use of anthelmintics. The growing emergence of resistant strains of *H. contortus* has resulted in the need to find new ways to prevent and control this parasite. So far, only one commercial vaccine was used in prevention the infection with this parasite [6]. More work should be done on exploring new antigens and further study needed on the biological characteristics of the known proteins [7–9].

Aspartyl protease inhibitors (API) are thought to protect intestinal parasitic nematodes from their hostile proteolytic environment; their presence could explain the resistance of the parasite to the digestive enzymes of the host [10]. In free-living life-cycle stages of parasitic or non-parasitic nematodes, API can also regulate endogenously secreted proteases [11]. Aspins from *Ostertagia ostertagi* [10], *Ascaris suum* [12], *Brugia malayi* [13] and *Parelaphostrongylus tenuis* [14] were found to be important in nematode infection.

In a recent research, proteins extracted from L3 and activated L3 (xL3) of *H. contortus* were analyzed by comparative proteomics. One hundred and twenty four protein spots were found to be differentially expressed. In that study, aspartyl protease inhibitor was identified and was found to be downregulated in xL3 when compared with free-living L3 [15]. However, the functions of this protein were not clear. In the present study, the gene encoding *H. contortus* was cloned and part of the biological characters of this protein was studied for the first time.

## Methods

### Animals, parasites and cells

Local 3–6 month-old goats were housed indoors and dewormed twice at 2 weekly intervals with levamisole (8 mg/kg body weight). Fecal sample from each goat was examined by microscopy for helminth eggs after 2 weeks. The animals excreting no eggs were used in the subsequent study.

SD rats (body weight ~150 g) were purchased from the Experimental Animal Center of Jiangsu, P. R. China (Qualified Certificate: SCXK 2008–0004). The animals were raised in a sterilized room and fed sterilized food and water.

*Haemonchus contortus* strain was kept in the laboratory of veterinary parasitology, Nanjing Agricultural University. Worms were maintained by serial passage in helminth-free goats as described before [16]. The procedures of collection and preservation of eggs, L3, xL3 and male and female adults of *H. contortus* were performed as described previously [17]. The isolation and culture of goat PBMCs were performed as described in [18].

### Cloning and sequence analysis of *API* gene

Total RNA was isolated from *H. contortus* adults. The DNA fragment encoding API was amplified by RT-PCR, with a pair of gene-specific primers. For the subsequent cloning, two enzyme restriction sites (*BamH*I and *Hind*III) were added at the 5′-end of the primers. The sequences of these primers are listed in Additional file 1: Table S1. After RT-PCR amplification, the products were purified by 1% agarose gel electrophoresis and cloned into pMD18-T vector (TaKaRa, Daliang, China). The recombinant plasmid pMD-18/API was transformed into *Escherichia coli* strain DH5α, cultured in Luria Bertini medium (LB) with ampicillin (100 μg/ml). The *API* gene was validated by sequence analyzing, and comparing online using the Blast program (http://www.ncbi.nlm.nih.gov).

### Expression and purification of recombinant API protein

The identified recombinant plasmids pMD-18/API was digested with restriction enzymes *BamH*I and *Hind*III. The target fragment of *API* was purified and cloned into the pET32a (+) expression plasmid vector digested with the same enzymes. The recombinant plasmid pET32/API was transformed into *E. coli* strain BL21 (DE3). Positive clones were picked out and cultured in LB with ampicillin (100 μg/ml) at 37 °C until OD_600_ achieved 0.6 [19]. Isopropyl-B-D- thiogalactopyranoside (IPTG) was added to the final concentration of 0.8 mM and incubated for another 5 h. To harvest the recombinant protein, the cell pellet was lysed using lysozyme (10 μg/ml) followed by sonication and then the cell lysates were analyzed by 12% (w/v) SDS-PAGE.

The recombinant API protein was purified by Ni^2+^-nitrilotriacetic acid (Ni-NTA) column according to the manufacturer’s instructions. The purified protein was refolded by renaturation buffer (20 mmol/l Tris-Cl, 500 mmol/l NaCl, 1 mmol/l GSH, 0.1 mmol/l GSSG, pH 8.0) containing different concentrations of urea (8, 6, 4, 2, 0 M) [20]. The concentration of refolded protein was determined according to the Bradford procedure [21].

About 0.3 mg of the purified recombinant API protein was mixed with Freund’s complete adjuvant as 1:1 mixture and inject into SD rats subcutaneously in multiple places as described by Han et al. [22]. Rats were boosted four times at two weeks intervals with the same dose of recombinant protein mixed with Freund’s incomplete adjuvant as 1:1 mixture. The sera against API were collected 10 days after the last immunization and stored at −80 °C until use. The serum against *H. contortus* was harvested from naturally infected goat [23].

The recombinant API protein and soluble proteins from *H. contortus* adult worms were separated by 12% SDS-PAGE, followed by electro-transferred onto a nitrocellulose membranes. After being blocked with 5% (w/v) skimmed milk powder in PBST (PBS with 0.5% Tween-20) at 4 °C overnight, the membranes were incubating with the first antibody (anti-*H. contortus* serum from goats or serum against recombinant API from rats) (1:1,000 dilution) for 1 h at 37 °C. Then, the membrane was washed three times with PBST and incubated with the secondary antibody (rabbit anti goat IgG-HRP or goat anti rat IgG-HRP (Santa Cruz Biotechnology, Dallas, USA) in PBST (1:5,000 dilution) for 1 h at 37 °C. After three washes, the immunoreaction was visualized using freshly prepared diaminobenzidine (DAB, Sigma Immuno-Chemicals, Dorset, UK) as a substrate for 5 min.

### Localization analysis

Freshly collected adult male and female *H. contortus* parasites were washed in PBS. The worms were dehydrated and then embedded in TISSUE-TEK® O.C.T. compound (SAKURA Finetek, Torrance, USA). They were snap-frozen in liquid nitrogen and stored at -20 °C until further processing. Using a cryotome (CM1950, Leica Instruments GmbH, Wetzlar, Germany), 8 μm thick sections were cut and mounted on Poly-L-lysine hydrobromide glass slides. The sections were further treated as described previously [17]. Briefly, sections were washed with PBS and blocked with 5% BSA in PBST for 1 h at 37 °C to prevent non-specific binding. Thereafter, the sections were incubated with rat anti-API serum (1:300 dilution) or normal rat serum (negative control) for 1 h at 37 °C. Following by washing with PBS for 3 times, the sections were incubated with Cy3-labeled Goat Anti-Rat IgG (1:1,000 dilution, Beyotime Institute of Biotechnology, Shanghai, China). After washing with PBS, the sections were immersed with Antifade Mounting Medium (Beyotime Institute of Biotechnology, Shanghai, China) which prevents the fading of fluorescence during microscopic examination.

### Differential expressions

Eggs, L3, xL3, male and female adult *H. contortus* were used for the differential expression of *API*. Total RNA was extracted and cDNA was reverse-transcribed using a HiScript® Q RT SuperMix for qPCR (Vazyme, Nanjing, China) according to the manufacturer’s instructions. Real-time PCR was performed on ABI 7500 Real Time PCR System (Applied Biosystems, New York, USA), using the standard procedure. The reactions and conditions of real time PCR are detailed in Additional file 2: Protocols. The primers for real-time PCR are listed in Additional file 1: Table S2. The amplification efficiencies of target and reference genes were verified and the results are shown in Additional file 3: Figure S1. The transcription levels for *API* in different life-cycle stages were analyzed by the ABI Prism 7500 software version 2.0.6 (Applied Biosystems, New York, USA) used the comparative Ct (2^−ΔΔCt^) method [24]. This experiment was repeated three times.

### Pepsin inhibition assay

Activity of the recombinant API protein in inhibiting protease (pepsin) was tested as described previously [25]. Fifty micrograms of recombinant API and the same weight of pepsin (Ameresco, Kaysville, USA) were dissolved in 10 mM acetate (pH 2.0) and incubated for 30 min at 37 °C, the residual enzyme activity of pepsin was determined by Pepsin assay kit (Nanjing Jiancheng Bio, Nanjing, China) according to the manufacturer’s instructions. Breifly, 150 μg of hemoglobin (Nanjing Jiancheng Bio, Nanjing, China) dissolved in the same buffer, was added to the above reaction and incubated for another 30 min at 37 °C and the resulting products were analyzed by 12% SDS-PAGE. The inhibition efficiencies for API in different reactive pH (2–10) and temperature (37–100 °C) were also checked. Trypsin (Ameresco, Kaysville, USA) was chosen as a control in this study. Each experiment was run in triplicate.

### Cytokine expressions in vitro

Effects of the recombinant API on the cytokine expression were evaluated. For that purpose goat PBMCs (1 × 10^8^) were incubated with 10, 20 and 40 μg of recombinant API or vehicle (PBS, negative control) for 12 h. Then, the cells were harvested and total RNA extracted. The transcriptions of *IL-2*, *IL-4*, *IL-10*, *IL-17*, *IFN-γ* and *TGF-β* were quantified by real time PCR. The reactions and conditions are described in Additional file 2: Protocols. The primers for real time PCR are listed in Additional file 1: Table S3. The amplification efficiencies of target and reference genes were verified and the results were shown in Additional file 3: Figure S2.

### Statistical analysis

In all experiments, data points were plotted using GraphPad Prism 6.0 (GraphPad Software, Inc., San Diego, CA). The data were presented as the mean and standard error of the mean (SEM) of three independent experiments. Real time PCR for cytokine expression analyses were performed using the Student’s *t*-test. *P*-value ≤ 0.05 was considered statistically significant.

## Results

### Cloning and expression of *API* from *H. contortus*

The complete coding sequence of *API* contains 681 bp was cloned from *H. contortus*, which encoded a protein of 226 amino acids with predicted molecular weight about 25 kDa and calculated pI of 5.95. The gene sequence was submitted to GenBank under accession number KY284864. The amino acid sequence of this protein was compared with the known aspins; this showed significant homology with aspin sequences from *O. ostertagi* (GenBank accession no. CAD10783, 91% identity), *T. colubriformis* (AY189824, 85% identity) and *C. elegans* (AAC46663, 51% identity).

**Fig. 1:**
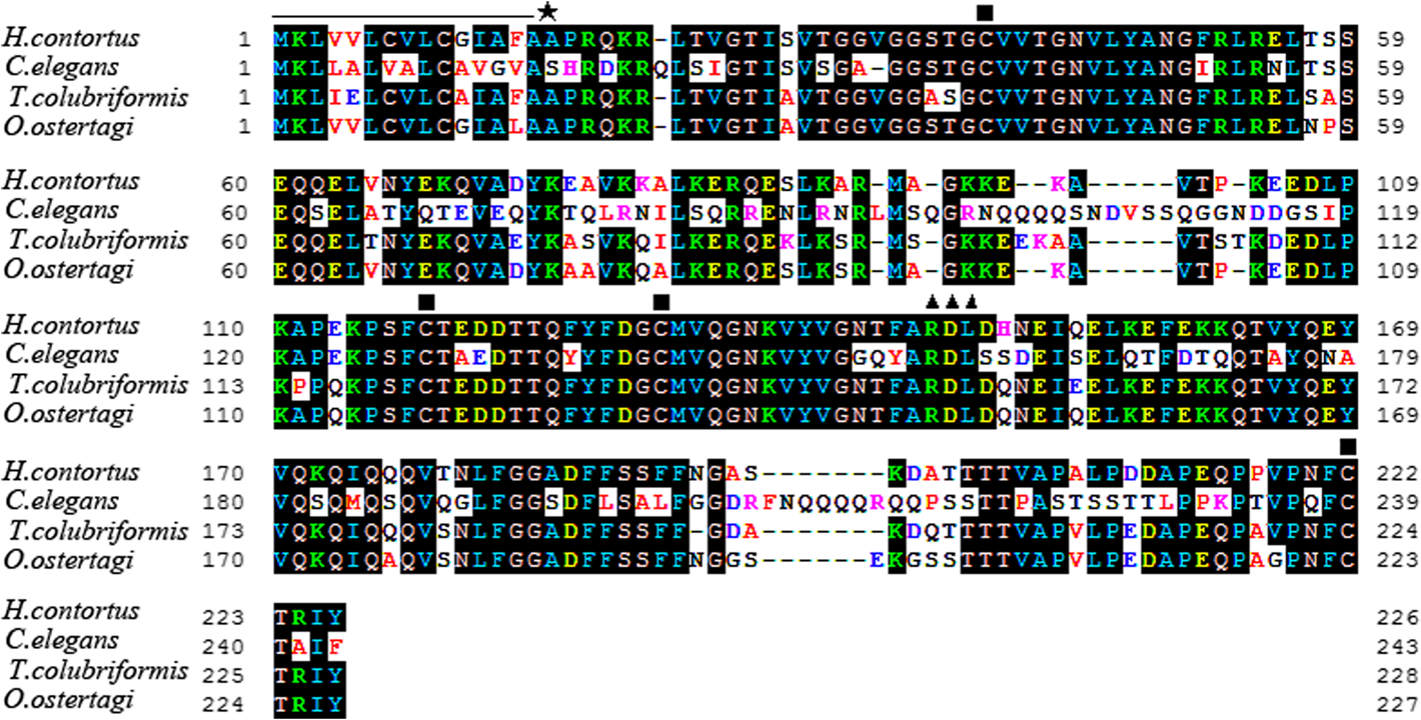
Alignment of *H. contortus* API with aspins from other nematodes. The amino acid sequence of *H. contortus* API is compared with those from *C. elegans* (AAC46663), *T. colubriformis* (AY189824) and *O. ostertagi* (CAD10783). The conserved cysteine residues and cleavage sites are marked by ■ and ★. “RDL” motif is indicated by ▲. Putative signal sequence is overlined

The alignment of *H. contortus* API with other nematode aspins is presented in Fig. 1. Some structures conserved in the aspin family were found in *H. contortus* API. There are four cysteine residues in the sequence. A predicted cleavage site between residues alanine (15) and alanine (16) was found at the N-terminal of the protein. The motif “YVRDLT” considered to be the active site of the aspartyl protease inhibitor was also found in this protein.

**Fig. 2:**
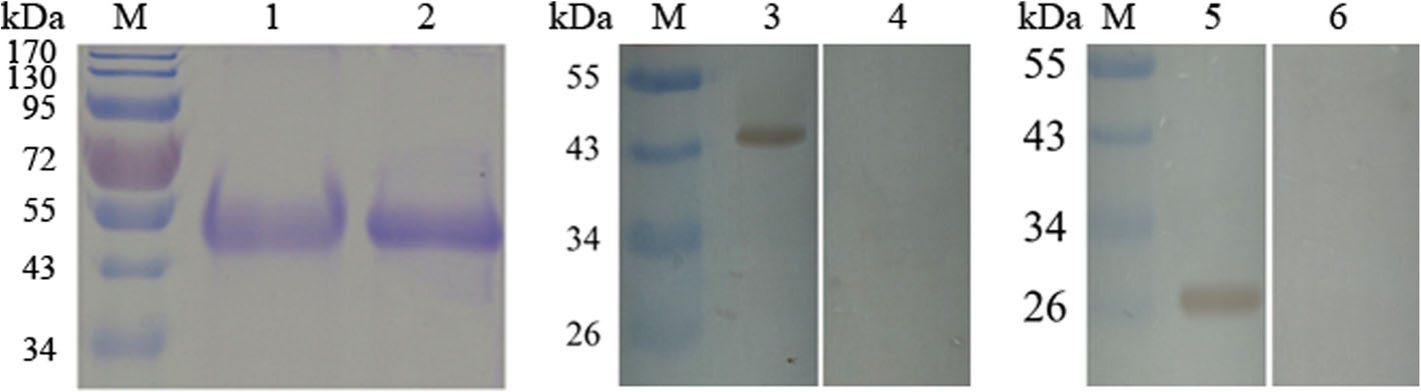
Purification of recombinant API protein and western blot. Recombinant API from the supernatants (Lane 1) and the inclusion body (Lane 2) of the bacterial cells were purified and separated by SDS-PAGE. Recombinant API was detected by serum from goat infected with *H. contortus* (Lane 3), and normal goat serum as negative control (Lane 4). API extracted from *H. contortus* was detected by rat antibody against recombinant API protein (Lane 5), and normal rat serum as negative control (Lane 6). Lane M: pre-stained protein ladder

The recombinant API was expressed as a fusion protein with the molecular weight of 43 kDa. The target protein was purified from the cell supernatants as well as inclusion body (Fig. 2, Lanes 1 and 2). Western blot showed that the recombinant API could be recognized by serum from goats infected with *H. contortus*, and the native API protein from *H. contortus* could be recognized by antibody against recombinant API (Fig. 2, Lanes 3 and 5). No protein was recognized by the normal serum (Fig. 2, Lanes 4 and 6).

### Localization of API

**Fig. 3:**
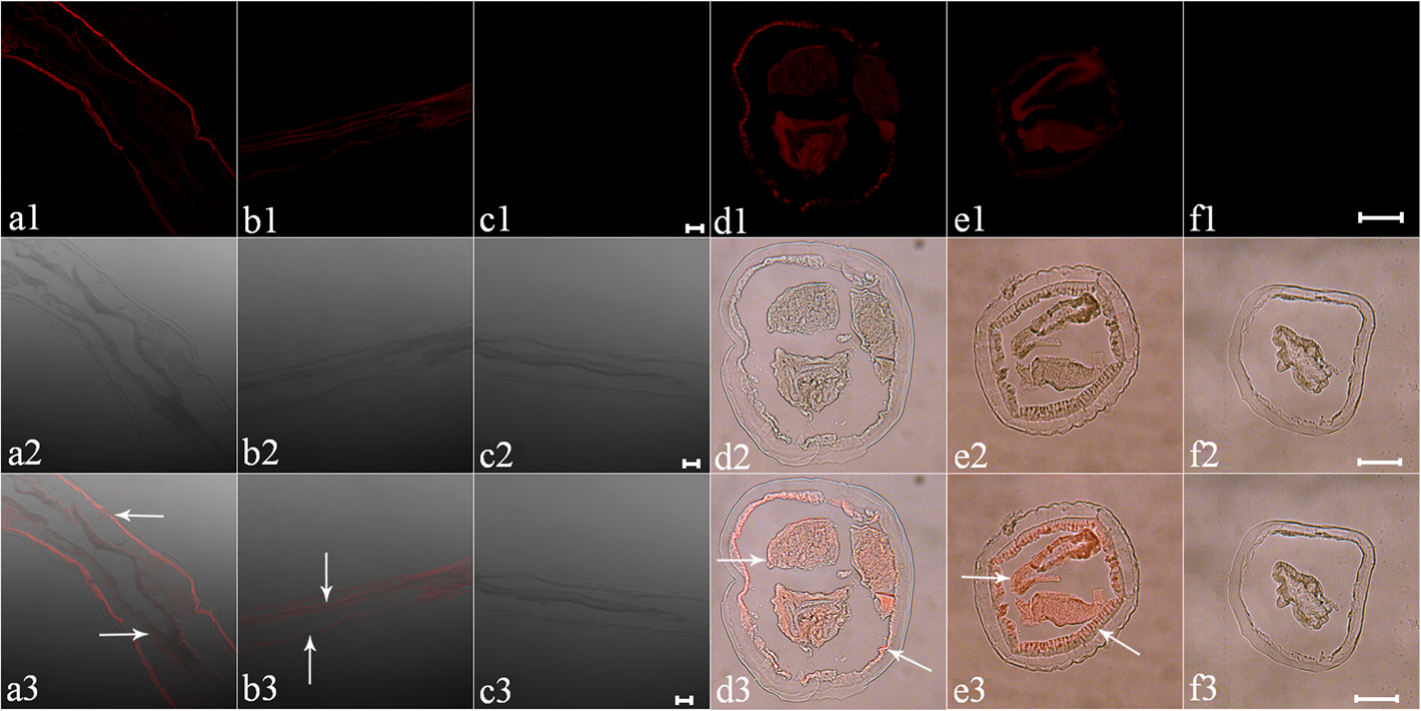
Localization of API in adult worms. Cryostal sections of *H. contortus* were incubated with rat antibody against the recombinant API, following with the second antibody Cy3-labeled Goat Anti-Rat IgG. The *red* fluorescence was detected in both female (**a**, **d**) and male (**b**, **e**) adult *H. contortus*, but no fluorescence was observed in control experiments (**c**, **f**). To see the distributions of the API, vertical (**a**, **b**, **c**) and transverse sections (**d**, **e**, **f**) were checked. Images in lines 1 and 2 were taken under UV light and white field; merged images are given in line 3. *Scale-bars*: 100 μm

Immunohistochemical test was performed to find out the native HcAPI localization site within the worms. Red specific fluorescence was detected on the internal surface and the gut of worms in the sections of both female and male adult *H. contortus*, whereas no red fluorescence was observed in controls (Fig. 3).

### Differential expression of *API*

**Fig. 4:**
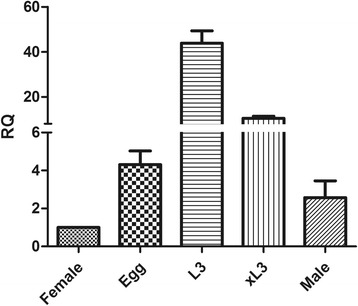
Expression patterns of *API* in different life stages of *H. contortus*. Expression levels of *API* mRNA in egg, L3, xL3, adult male and female were tested by real time PCR. The relative quantities (RQ, compared with female adult worm, female = 1) in egg, L3, xL3 and adult male were 4.3, 43.8, 10.5 and 2.6, respectively. The value of *API* gene in the female was normalized to 1.0. The relative changes in gene expression ratios of selected genes were normalized to the expression of a single reference gene and calculated as described by the 2^-ΔΔCt^ method. Error bars indicate SEM from three independent experiments

The results of real time PCR indicated that transcriptions of *API* were significantly different in the eggs, L3, xL3, adult male and female of *H. contortus* (Fig. 4). The highest expression of *API* was found in the free living stage (L3). When L3 was activated and turned into parasitic stage xL3, the expressions were downregulated, and the expression levels continuously decreased in the adults.

### Pepsin inhibition assay

**Fig. 5:**
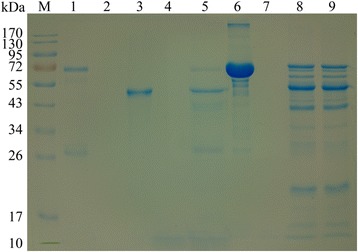
Inhibition assay of API on pepsin and trypsin. The products of hemoglobin (BSA) digested by pepsin (trypsin) with (or without) API were analysed by SDS-PAGE. Lane 1: hemoglobin; Lane 2: pepsin; Lane 3: API; Lane 4: pepsin + hemoglobin; Lane 5, pepsin incubated with API for 30 min (at 37 °C, pH 4.0) + hemoglobin; Lane 6: BSA; Lane 7: trypsin; Lane 8: trypsin + BSA; Lane 9: trypsin incubated with API for 30 min (at 37 °C, pH 4.0) + BSA

**Fig. 6:**
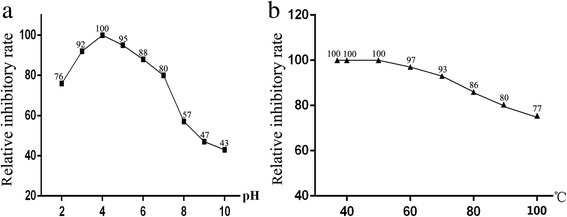
Effects of pH value and temperature on the inhibition activities of recombinant API. Recombinant API was dissolved in the buffer with a serial pH value from 2 to 10 for 30 min (**a**), or treated at temperatures between 37 and 100 °C (**b**). The result API was added to the reaction with Pepsin and hemoglobin, and the relative inhibitory rate was tested by pepsin assay kit. The inhibition activity was defined as 100 when the reaction was carried out under pH 4.0 at 37 °C

As shown in Fig. 5, the inhibition activity of the recombinant API was measured. The activity of pepsin in hemoglobin digestion declined significantly when compared with the control (Lanes 4 and 5). However, the activity of trypsin in BSA digestion was not inhibited by this recombinant API (Lanes 8 and 9). To quantitate the inhibition effects of the recombinant API, a pepsin assay kit was used. The results showed that the activity of pepsin was decreased by 63% when compared with the negative control (without adding the recombinant API). It was found that the recombinant API showed the best inhibition effects at the pH of 4.0 (Fig. 6a) at 37–50 °C (Fig. 6b). The recombinant API also showed partial inhibition activities (relative inhibitory rate > 50%) under pH between 2.0 and 8.0 (Fig. 6a). The recombinant API protein kept stable at temperatures between 37 and 80 °C (Fig. 6b).

### Effects on cytokine expression by goat PBMCs

**Fig. 7:**
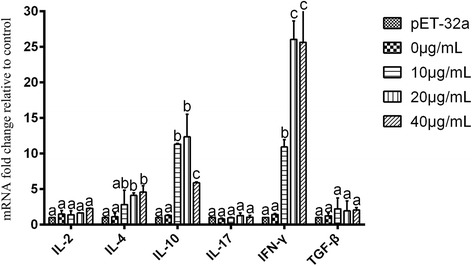
Levels of multiple cytokines stimulated by the recombinant API. Goat PBMCs were incubated with the recombinant API for 12 h, the mRNAs encoding IL-2, IL-4, IL-10, IL-17, IFN-γ and TGF-β were quantified by real time PCR. Different letters (a, b and c) above the error bars indicate significant differences (*P* < 0.05)

PBMCs consist of several populations of T cells, B cells, NK cells and other monocytes that play important roles in the immune responses. In the present study, in vitro effects of the recombinant API protein on the cytokine expression in PBMCs were evaluated. Result of transcription levels of *IL-2*, *IL-4*, *IL-10*, *IL-17*, *IFN-γ* and *TGF-β* are shown in Fig. 7. After incubation with the recombinant API, the expressions of *IFN-γ* (*t*_(4)_ = 13.74, *P* = 0.0048), *IL-4* (*t*_(4)_ = 5.17, *P* = 0.034) and *IL-10* (*t*_(4)_ = 4.35, *P* = 0.048) increased significantly, while those of *IL-2* (*t*_(4)_ = 1.55, *P* = 0.26), *IL-17* (*t*_(4)_ = 1.36, *P* = 0.30) and *TGF-β* (*t*_(4)_ = 2.11, *P* = 0.12) were not significantly different.

## Discussion

Protease inhibitors have been isolated from a number of parasitic nematodes [26, 27], and partial characters of serpin and cystatin from *H. contortus* were studied [23, 28, 29]. However, no information of aspin was reported in *H. contortus*. To our knowledge, in this study an aspin gene from *H. contortus* was cloned, expressed and part its biological characteristics studied for the first time.

Studies indicated that the motif “YVRDLT” would be important for the activities of aspins [30]. However, this was not well conserved in API from *H. contortus*. The motif sequence was replaced by the shortened “RDL”. Similar results were also found in other nematodes [10, 31]. This may suggest that the “RDL” may be of crucial functional importance in inhibitory activities. It has been reported that API in different nematodes can inhibit the activity of pepsin and cathepsin E [25, 32]. In this study, the recombinant API from *H. contortus* showed the highest inhibitory activities in pepsin digestion of hemoglobin under the optimal conditions. The recombinant API showed partial inhibitory activities at different pH values and different temperatures. Even when the protein was treated at 100 °C, residual inhibitory activity remained (77%). Similar results were reported in *Coriolus versicolor* [33]; in that study, 7% of the inhibitory activity was lost after treatment of the aspartyl protease inhibitor at 98 °C. Recent studies may explain that protease inhibitors possess thermal stability were based on quantity of α-helix and β-pleated sheet in the secondary structure of the protein [34].

The presence of aspin homologues in the free-living nematode *C. elegans* suggests that these inhibitors could function in ways that are not related to the development of parasitism by inhibiting endogenous proteases [10]. Research on *O. ostertagi* suggested that API played a definite role in the pre-parasitic L3 stage of the parasite [9]. In this study, it was found that the expression of API decreased significantly from free living L3 to parasitic adults. Our western blot result showed that the recombinant API could be recognized by the antiserum from goats infected with *H. contortus*. This validated that API has been secreted by the worms and induced the immune responses of the host. A deduced signal sequence found in the N-terminal of API protein may support the idea that this protein could be secreted by the parasite. All of the results indicate that API may play important roles during the infection with *H. contortus.*

Immunohistochemical test is widely used in native antigen location and distribution in parasite research. In our study, the native API was detected on the internal surface and the gut of both adult male and female worms, thus we can consider it is an excretory secretory antigen which can secrete into host through the parasite cuticle or gut. In the early infection with *H. contortus*, complement fixation is one of the innate responses of the host immune system [35]. Delayed immunity of *H. contortus* is regulated by CD4+ T lymphocytes, IgA and IgE antibodies, eosinophil cytotoxicity and the classical complement pathway. Th2 response is a part of the humoral response associated with helminths and is characterized by the secretion of the IL-4, IL-5 and IL-10 [36, 37].

Immunosuppressive cytokine IL-10 produced by inducible T regulatory cells (Treg) was able to inhibit the development of allergic Th2 cell responses [38–40]. In our study, we found that recombinant API proteins could enhance goat PBMCs to produce IL-10. This means that this protein could induce the Treg cells and thus facilitate the worm infection.

IL-4 is a key regulator in humoral immunity, induces B-cell class switching to IgE and regulates MHC class II production [41, 42]. In the present study, recombinant API increased the production of IL-4, whereas IL-2, IL-17 and TGF-β were not affected. This may indicate that this protein might be a protective antigen in the immune response against the infection with *H. contortus*.

Cytokine-regulated cellular immunity is important for host defense mechanisms, Th1 cells produce pro-inflammatory cytokine IFN-γ and regulate antigen presentation and cellular immunity against infection [43–45]. In the present study, we observed the transcription of IFN-γ was increased in PBMCs incubated with API. The results indicate that API protein might play a role in the immunization against *H. contortus*. However, the IFN-γ could inhibit the development of Th2 cells in the immune responses [46]. Thus, the real effects of the enhanced IFN-γ on the resistance of the goats to *H. contortus* should be further investigated.

## Conclusion

The present results indicate that API may play important roles during the infection with *H. contortus.* The western blot indicated that the recombinant API could be recognized by the antisera from goats infected with *H. contortus*. This means API has been secreted by the worms and could regulate the immune responses of the host by modulating the various cytokines. However, these results were observed from in vitro experiments; the real functions of API should be further investigated in vivo.

## Additional files

Additional file 1: Table S1. Primers for amplification of *API* gene. **Table S2.** Primers for real time PCR. **Table S3.** Primers for quantification of cytokines transcription. (DOCX 18 kb)

Additional file 2: Protocols. (DOCX 15 kb)

Additional file 3: Figure S1. Amplification efficiencies of target gene (*API*) and endogenous reference (*β-Tubulin*) gene were verified to be similar by real time PCR. **Figure S2.** Amplification efficiencies of target genes (*IL-2*, *IL-4*, *IL-10*, *Il-17*, *IFN-γ* and *TGF-β*) and endogenous reference (*β-Actin*) gene were verified to be similar by real time PCR. (DOCX 57 kb)

## Abbreviations

2D-DIGE: Two-dimensional differential gel electrophoresis
API: Aspartyl protease inhibitor
Aspin: Aspartyl protease inhibitor
Cy3: Cyanine dyes 3
DAB: Diaminobenzidine
IFN-γ: Interferon-γ
IgG-HRP: Horseradish peroxidase labeled immunoglobulin G
IL-10: Interleukin-10
IL-17: Interleukin-17
IL-2: Interleukin-2
IL-4: Interleukin-4
IPTG: Isopropyl-B-D-thiogalactopyranoside
L3: Third-stage larva
LB: Luria Bertini medium
Ni-NTA: Ni^2+^ - nitrilotriacetic acid
OCT: Optimal cutting temperature
PBMCs: Peripheral blood mononuclear cells
PBS: Phosphate buffered saline
SDS-PAGE: Sodium dodecyl sulfate polyacrylamide gel electrophoresis
TGF-β: Transforming growth factor-β
