# Supplementary material for: Characterization of a novel aspartyl protease inhibitor from Haemonchus contortus
Source: Parasit Vectors. 2017 Apr 19;10:191. doi: 10.1186/s13071-017-2137-1 (PMC5395858; doi:10.1186/s13071-017-2137-1)
Supplement: Supplementary file 1 — Primers for amplification of API gene. Table S2. Primers for real time PCR. Table S3. Primers for quantification of cytokines transcription. (DOCX 18 kb) [file 13071_2017_2137_MOESM1_ESM.docx]

**Table S1. Primers for amplification of *API* gene**

| Gene Name | Primer Sequence (5’-3’) |
| --- | --- |
| *API* | CGCGGATCCATGAAGTTGGTCGTGCTCTGTG |
|  | CCCAAGCTTTCAATAGATTCTCGTACAGAAGTTAGG |

**Table S2. Primers for real time PCR**

| Gene Name | Primer Sequence (5’-3’) |
| --- | --- |
| *β-Tubulin* | TGCTATGTTCCGTGGTCGTA |
|  | GCAGTGAATTGCTCCGAAAT |
| *API* | ACACCCAAGGAGGAGGATCT |
|  | TTCAGCTCCTGGATTTCGTT |

**Table S3. Primers for quantification of cytokines transcription**

| Gene Name | Primer Sequence (5’-3’) |
| --- | --- |
| *β-Actin* | CACCACACCTTCTACAAC |
|  | TCTGGGTCATCTTCTCAC |
| *IL-2* | CAAACGGTGCACCTACTTCA |
|  | AGCTTGAGGTTCTCGGGATT |
| *IL-4* | GTACCAGCCACTTCGTCCAT |
|  | GCTGCTGAGATTCCTGTCAA |
| *IL-10* | CCTTGTCGGAAATGATCCAG |
|  | AGGGCAGAAAACGATGACAG |
| *IL-17* | TTGTAAAGGCAGGGGTCATC |
|  | GGTGGAGCGCTTGTGATAAT |
| *IFN-γ* | GAACGGCAGCTCTGAGAAAC |
|  | GGTTAGATTTTGGCGACAGG |
| *TGF-β* | GAACTGCTGTGTTCGTCAGC |
|  | TCCAGGCTCCAGATGTAAGG |
