# Supplementary material for: Characterization of a novel aspartyl protease inhibitor from Haemonchus contortus
Source: Parasit Vectors. 2017 Apr 19;10:191. doi: 10.1186/s13071-017-2137-1 (PMC5395858; doi:10.1186/s13071-017-2137-1)
Supplement: Supplementary file 2 — Protocols. (DOCX 15 kb) [file 13071_2017_2137_MOESM2_ESM.docx]

**Protocols**

The real time PCR reactions were carried out in 96-well optical reaction plates (Bio-Rad Laboratories , Hercules，California，USA) with 200 nM of each specific primer, 2 μl cDNA (20 ng, as described in the experimental procedures), and HiScript^®^ Q RT SuperMix for qPCR (Vazyme, China) using an ABI 7500 Real-Time PCR system (Applied Biosystems, Foster City, California, USA). The PCR were performed as follows: 95°C for 30 sec, followed by 40 cycles of 95°C for 15 sec, 60°C for 30 sec and 72°C for 30 sec.
