# Supplementary material for: Characterization of a novel aspartyl protease inhibitor from Haemonchus contortus
Source: Parasit Vectors. 2017 Apr 19;10:191. doi: 10.1186/s13071-017-2137-1 (PMC5395858; doi:10.1186/s13071-017-2137-1)
Supplement: Supplementary file 3 — Amplification efficiencies of target gene (API) and endogenous reference (β-Tubulin) gene were verified to be similar by real time PCR. Figure S2. Amplification efficiencies of target genes (IL-2, IL-4, IL-10, Il-17, IFN-γ and TGF-β) and endogenous reference (β-Actin) gene were verified to be similar by real time PCR. (DOCX 57 kb) [file 13071_2017_2137_MOESM3_ESM.docx]

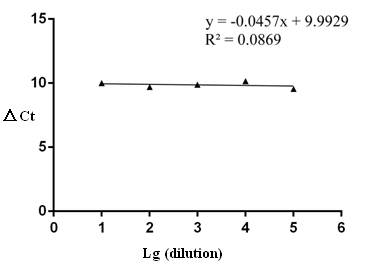


**Figure S1. Amplification efficiencies of target (*API*) and endogenous reference (*β-Tubulin*) genes were verified to be similar by real time PCR.**


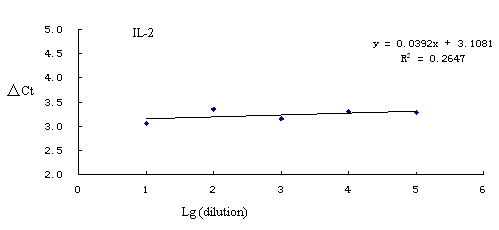


**Figure S2-1. Amplification efficiencies of target (*IL-2*) and endogenous reference (*β-Actin*) genes were verified to be similar by real time PCR.**


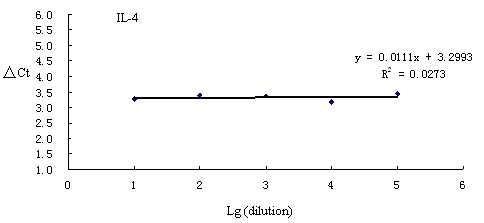


**Figure S2-2. Amplification efficiencies of target (*IL-4*) and endogenous reference (*β-Actin*) genes were verified to be similar by real time PCR.**


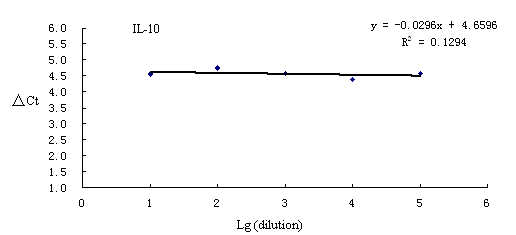


**Figure S2-3. Amplification efficiencies of target (*IL-10*) and endogenous reference (*β-Actin*) genes were verified to be similar by real time PCR.**


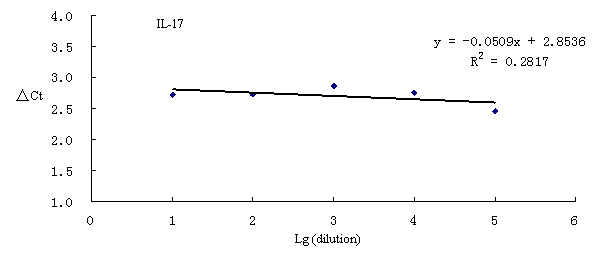


**Figure S2-4. Amplification efficiencies of target (*IL-17*) and endogenous reference (*β-Actin*) genes were verified to be similar by real time PCR.**


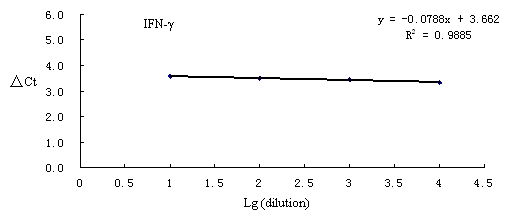


**Figure S2-5. Amplification efficiencies of target (*IFN-γ*) and endogenous reference (*β-Actin*) genes were verified to be similar by real time PCR.**


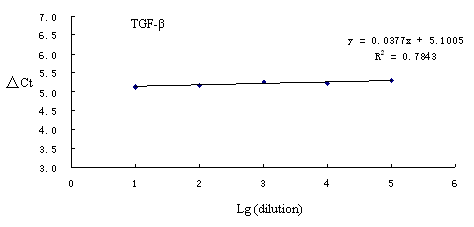


**Figure S2-6. Amplification efficiencies of target (*TGF-β*) and endogenous reference (*β-Actin*) genes were verified to be similar by real time PCR.**
